# Scaffold proteins as dynamic integrators of biological processes

**DOI:** 10.1016/j.jbc.2022.102628

**Published:** 2022-10-20

**Authors:** Christopher J. DiRusso, Maryam Dashtiahangar, Thomas D. Gilmore

**Affiliations:** Department of Biology, Boston University, Boston, Massachusetts, USA

**Keywords:** scaffold protein, conformational change, signaling, KSR, NEMO, SHANK3, STE5, liquid–liquid phase separation, IDR, intrinsically disordered region, IVD, intervening domain, LLPS, liquid–liquid phase separation, PSD, postsynaptic density, SAXS, small angle X-ray scattering, XRC, X-ray crystallography, ZF, zinc finger

## Abstract

Scaffold proteins act as molecular hubs for the docking of multiple proteins to organize efficient functional units for signaling cascades. Over 300 human proteins have been characterized as scaffolds, acting in a variety of signaling pathways. While the term scaffold implies a static, supportive platform, it is now clear that scaffolds are not simply inert docking stations but can undergo conformational changes that affect their dependent signaling pathways. In this review, we catalog scaffold proteins that have been shown to undergo actionable conformational changes, with a focus on the role that conformational change plays in the activity of the classic yeast scaffold STE5, as well as three human scaffold proteins (KSR, NEMO, SHANK3) that are integral to well-known signaling pathways (RAS, NF-κB, postsynaptic density). We also discuss scaffold protein conformational changes vis-à-vis liquid–liquid phase separation. Changes in scaffold structure have also been implicated in human disease, and we discuss how aberrant conformational changes may be involved in disease-related dysregulation of scaffold and signaling functions. Finally, we discuss how understanding these conformational dynamics will provide insight into the flexibility of signaling cascades and may enhance our ability to treat scaffold-associated diseases.

Intracellular signaling cascades are complex processes that require precise spatiotemporal control (1). From the initial binding of a ligand to a cell surface receptor to the downstream gene expression changes required for many biological responses, the cell often coordinates complex molecular events *via* control boards that organize the timing and strength of such signals and the array of proteins involved.

The term scaffold has been used in several ways, but the general consensus is that a scaffold is a protein that binds two or more proteins to increase the efficiency of a molecular event, often one that is involved in signal transduction (2). As such, scaffolds serve as docking platforms, which act as switchboards to reduce the chaos of the cellular “soup.” Thus, the primary function of scaffolds is to overcome the difficulty of organizing complex signal transduction and feedback mechanisms within a cell by regulating these molecular events in time and space. As such, scaffolds provide the advantage of modular regulation in that they are responsible for rapid mobilization of signaling components based on external signals. By organizing a set of master controlling molecules such as scaffolds, instead of the larger pool of signaling enzymes, a cell can increase the flexibility of signaling while minimizing energy expenditure. In this way, scaffolds ensure that proper responses occur in either one or multiple signaling pathways. Overall, scaffolds comprise a class of proteins that are central to enhancing signaling cascades, have interaction domains such that they can interact with multiple binding partners, facilitate higher order complex formation, and are often highly conserved (3, 4).

Even though they may be catalytically inactive, scaffolds are dynamic in their mechanism of action (5, 6, 7). As such, the term scaffold has recently taken on new connotations, especially in the field of conformational change and liquid–liquid phase separation (LLPS). Therefore, it is important to rethink how scaffolds function in a dynamic cellular context and how conformational changes in scaffolds are central to the regulation of signaling pathways and molecular processes.

In this review, we present a critical analysis of scaffolds as plastic regulators of signaling cascades and provide examples of how conformational changes within scaffolds can impact their dynamic roles in signal transduction. We catalog conformational changes in scaffold proteins and describe examples of how conformational flexibility imparts a regulatory property onto several well studied scaffolds. We also describe what is known about scaffold dysfunction in human disease and how the understanding of scaffold dynamics may be applied to the modulation of scaffold function and new disease therapies.

## Scaffolds, conformational changes, and disease associations

To comprehensively search for scaffolds that have been reported to undergo conformational changes, we used a human scaffold database (ScaPD) (8), as well as an ad hoc literature search. From this, we identified 35 human scaffolds (20 out of 291 from ScaPD and 15 from our manual literature search) that have been documented to undergo conformational changes during signaling (Table 1). These 35 scaffolds are part of many signaling pathways, including ones involved in cell proliferation, cell survival and cell death, cell adhesion and motility, neural function, immunity, and differentiation. For most scaffolds in Table 1, conformational changes have been documented by structural approaches, including X-ray crystallography (XRC), NMR, small angle X-ray scattering (SAXS), and cryo-EM, or by computational modeling based on existing crystal structures. For others, biochemical or biophysical methods strongly suggest conformational changes, such as for the exposure of protein binding domains or altered mobility during electrophoresis. It is likely that the number of scaffolds with validated conformational changes will continue to increase.

**Table 1:** Scaffold proteins that undergo conformational change during signaling and diseases associated with scaffold protein mutations^a^

Scaffold^b^	Signaling pathway	Scaffold-associated diseases (mutation effect)^c^	GeneCards identifier	Conformational change validation method^d^
*Cell cycle*				
AKAP18	cAMP, PKA, and Ca^+2^ signaling	Febrile seizures, Familial, 7; gestational diabetes insipidus (LOF)	GC06P131126	XRC, EM (81, 82)
AKAP13	cAMP, PKA, and Ca^+2^ signaling	Breast cancer; long Qt syndrome (GOF)	GC15P118052	XRC (83, 84, 85)
KSR2	MAPK	Type 2 diabetes; lung adenocarcinoma (LOF)	GC12M117453	XRC (23, 29)
*Cell proliferation*				
GRB2	RTK-mediated	Liver and lung cancer (GOF)	GC17M075318	ITC (86)
IRS-1	GH and IR	Type 2 diabetes; hypotrichosis 13 (LOF)	GC02M226731	Molecular modeling (87)
IQGAP1	Various; including: Hippo, RAF/MAP kinase PI3K/AKT, Wnt, TGF-β signaling	Bullous skin disease; gastric cancer (LOF)	GC15P090388	XRC (88)
SHC1	RTK-mediated	Multiple endocrine neoplasia, Type Iia; malignant astrocytoma (GOF)	GC01M154962	SAXS (89, 90)
SHOC2	MAPK	Noonan-like syndrome with loose anagen hair (GOF)	GC10P110919	XRC (91)
*Immunity*				
BCL10	BCR and TCR	Mucosa-associated lymphoma; mesothelioma (GOF)	GC01M085265	Cryo-EM/NMR (92, 93)
CARMA1	BCR and TCR	B-cell lymphoma (GOF)	GC07M002906	Biochemical (PPI) (94)
ITK	TCR-induced Ca^+2^ and NFAT signaling	Lymphoproliferative Syndrome 1; (LOF)	GC05P157158	NMR (95)
NEMO	NF-κB	Incontinentia Pigmenti; ectodermal dysplasia and immunodeficiency (LOF)	GC0XP154541	SAXS (41, 42)
VAV1	T-cell and B-cell signaling	Angioimmunoblastic T-cell lymphoma (GOF)	GC19P006772	XRC, NMR (96)
WASP	T-cell signaling	Wiskott-Aldrich syndrome; Thrombocytopenia 1; neutropenia, severe congenital, X-linked (LOF)	GC0XP048676	BiFC (97, 98)
*Cytoskeletal remodeling/adhesion/migration*				
CRK	Tyrosine kinase pathways	Chromosome 17P13.3 Duplication Syndrome (GOF)	GC17M001420	NMR (99, 100)
Dystrophin	Various; including muscle cell signaling	Duchenne muscular dystrophy (LOF)	GC0XM031097	SANS (101)
Ezrin	Cytoskeleton-mediated	Autosomal recessive nonsyndromic intellectual disability; neurofibromatosis, Type 2 (LOF)	GC06M158765	XRC (102, 103)
FAK	Cytoskeleton-mediated	Malignant astrocytoma; diffuse gastric cancer (GOF)	GC08M140657	FRET (104, 105)
FLNA	Cytoskeleton-mediated	Frontometaphyseal dysplasia (GOF)	GC0XM154348	Biochemical (Electrophoresis) (106)
BCAR1	Cytoskeleton-mediated	Antiestrogen resistance in breast cancer (LOF)	GC16M075228	AMF (107, 108)
β-Arrestin-2	GPCRs	Synovium neoplasm; cryptococcal meningitis; Autism (LOF)	GC17P004711	BRET (109)
*Neuronal Activity*				
DISC1	cAMP, PKA and Ca^+2^ signaling	Schizophrenia (LOF)	GC01P231626	Molecular modeling (110, 111)
JIP1	JNK and NF-κB	Type 2 diabetes; ischemia (LOF)	GC11P046501	XRC, NMR (112, 113)
PAK1	PDK1–Akt	Gastroesophageal junction adenocarcinoma (GOF)	GC11M087708	XRC (114)
SH3RF1	JNK and NF-κB	--		XRC, NMR (113)
PSD-95	Excitatory synaptic	Intellectual developmental disorder 62; cerebral degeneration (LOF)	GC17M007189	XRC, NMR, SAXS (115)
RGS4	GPCRs	Bipolar disorder; schizophrenia (LOF)	GC01P163038	XRC, TRFS (116, 117)
SHANK3	Excitatory synaptic	Schizophrenia 15; Phelan-Mcdermid syndrome; autism (LOF)	GC22P050674	XRC, Molecular modeling (48)
X11/Mint	NMDA receptor	Neuronal intranuclear inclusion disease; syndromic X-linked intellectual Disability Najm Type (LOF)	GC09M069427	NMR (118)
*Other biological processes*				
AP2	Clathrin-mediated endocytosis	Alzheimer’s disease (LOF)	GC11P000924	XRC (119, 120)
NBN	ATM	Nijmegen breakage syndrome; aplastic anemia (LOF)	GC08M089933	XRC, SAXS (121)
SLC9A3R1	Ezrin- associated GPCR signaling	Nephrolithiasis/osteoporosis, hypophosphatemic, 2 (LOF)	GC17P074749	NMR, SANS, MS (122, 123)
NHERF3/PDZK1	T-cell, B-cell, and integrin signaling	Gout; inflammatory diarrhea (LOF)	GC01M145670	Biochemical (Electrophoresis) (124)
SQSTM1/p62	TRAF6-dependent	Paget disease of Bone 3 (LOF)	GC05P179806	NMR (125, 126)
ZO-1	Wnt/β-catenin	Arrhythmogenic cardiomyopathy (LOF)	GC15M029699	SIM (127, 128)

a List was curated from ScaPD database as well as a manual literature search. Proteins are organized by best fit biological process.

b Abbreviations: AKAP, A-kinase anchoring protein 18; AKAP-Lbc, A-kinase-anchoring protein-Lbc; AP-1/AP-2, Adaptor Protein Complex 1/2; ATM, ataxia-telangiectasia mutated; BCL10, B cell lymphoma 10; BCR, B-cell receptor; CARMA1, Caspase recruitment domain-containing MAGUK protein 1; CRK, Chicken tumor virus number 10 regulator of kinase; DISK1, Disrupted-in-schizophrenia-1; eIF4G, Eukaryotic initiation factor 4 gamma; FAK, Focal adhesion kinase; FLNA, Filamin A; GPCR, G-protein coupled receptor; Grb2, Growth factor receptor bound protein 2; IGF-1, Insulin growth factor 1; IQGAP1, IQ motif containing GTPase activating protein 1; IRS-1, Insulin receptor substrate-1; ITK, IL2 inducible T cell kinase; JIP1, JNK-interacting protein; JNK, c-Jun N-terminal kinase; KSR2; kinase suppressor of RAS; MAPK, mitogen-activated protein kinase; NBS1, Nijmegen breakage syndrome 1; NEMO, NF-kappa-B essential modulator; NHERF1, Na+/H+ exchanger regulatory factor 1; NHERF3, Na+/H+ exchanger regulatory factor 3; p130Cas, Crk-associated substrate; NMDA, N-methyl-D-aspartate; PAK1, P21-activated protein kinase 1; PKA, protein kinase A; PKC, Protein kinase C; PSD-95, Postsynaptic density protein-95; RACK1, receptor for activated C kinases; RGS4, Regulator of G protein signaling 4;; RTK, receptor tyrosine kinase; SH3RF1, SH3 domains and ring finger; SHANK3, SH3 and multiple Ankyrin repeat domains 3; SHC1, SHC-transforming protein 1; SHOC-2, Leucine-rich repeat protein SHOC-2; SQSTM1, Sequestosome 1; TCR, T-cell receptor; VAV, Vav guanine nucleotide exchange factor 1; WASP, Wiskott–Aldrich syndrome protein; ZO1, zonula occludens-1.

c GOF, gain-of-function mutation; LOF, loss-of-function mutation.

d AMF, atomic force microscopy; BiFC, bimolecular fluorescence complementation; BRET, bioluminescence resonance energy transfer; EM, electron microscopy; ITC, isothermal titration calorimetry; MS, mass spectroscopy; PPI, protein–protein interaction; SANS, small angle neutron scattering; SAXS, small angle X-ray scattering; SIM, structured illumination microscopy; TRFS, time-resolved fluorescence spectroscopy.

Mutations in 34 of these 35 scaffolds have been directly linked to human disease. Such mutations affect the ability of the scaffold to bind a partner or render the scaffold unable to undergo proper conformational change upon substrate binding. Indeed, in at least two cases, disease mutations have been shown to affect a key conformational change in the scaffold. Namely, gain-of-function mutations in CARMA1 that disrupt its ability to undergo inhibitory conformational changes occur in some human B-cell lymphomas. Specifically, these mutations induce the stable conversion of CARMA1 to an open, active state, which promotes downstream signaling in the BCL10 pathway to chronically activate NF-κB, which blocks apoptosis and enhances proliferation (9). Similarly, some Wiskott–Aldrich syndrome patients have a missense loss-of-function mutation in WASP that prevents conformational changes crucial for WASP activity in T cells (10). A perturbed conformational change in WASP is also predicted to be involved in the development of X-linked severe congenital neutropenia (11).

Overall, it is clear that conformational change—and not simply protein docking—is critical for a growing number of scaffolds and is almost certainly involved in signaling in ways that are yet to be discovered. As such, it is likely that conformational change in scaffold proteins is important for a wide range of biological functions and human diseases.

## Conformational changes in four well-known scaffold proteins

In this section, we describe conformational changes that are involved in the activities of four well-characterized scaffolds, serving as a paradigm for how dynamic changes in scaffolds can affect the activities of molecular complexes and signaling pathways.

### STE5 in the control of yeast mating

The yeast protein STE5 is no doubt the best studied scaffold, and it is central to a kinase-based cascade required for yeast mating. Although it has been approximately 40 years since its discovery as a scaffold (12), STE5 continues to provide a framework for understanding scaffold proteins in other organisms, especially as related to conformational change.

**Figure 1:**
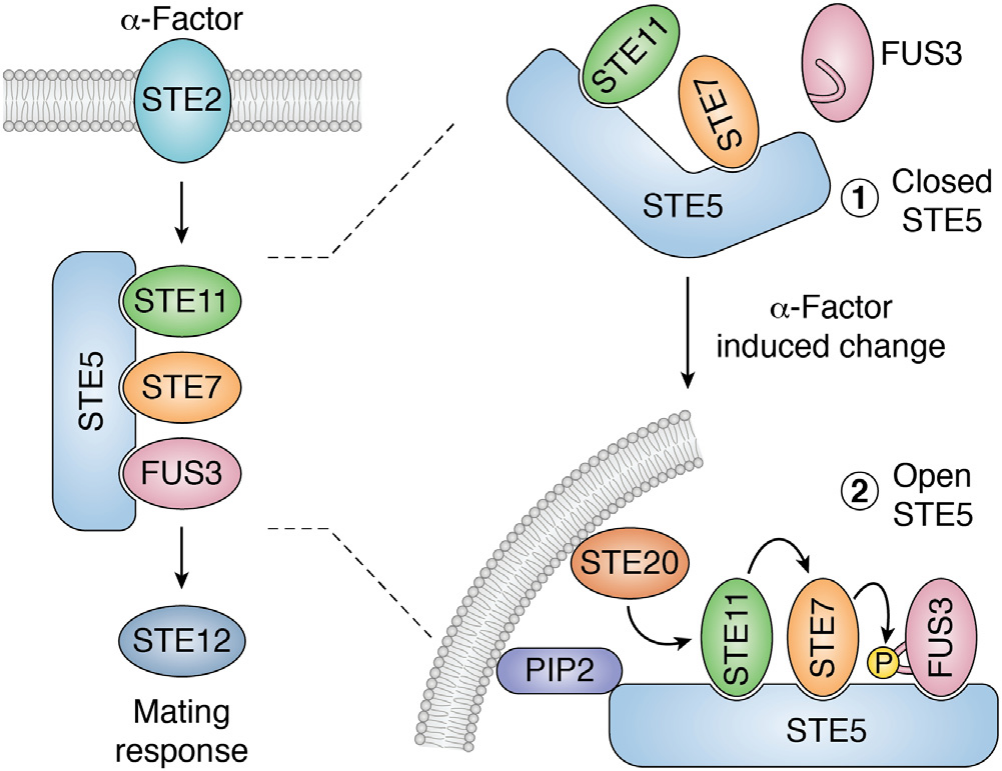
**Scaffold STE5 has pathway regulatory properties mediated *via* conformational changes**. On the left is the traditional stable platform depiction of the three-tiered MAPK cascade modulated by STE5 in yeast, where α-factor binds to STE2 and a phosphorylation cascade involving STE11, STE7, and FUS3 is initiated. This leads to the eventual phosphorylation of the STE12 transcription factor to initiate transcription of mating response genes. On right is a more detailed depiction of the complexity of STE5’s role in the pathway. In unstimulated cells, STE5 is in a closed state where FUS3 binding is blocked. After α-factor binds to its receptor STE2, STE5 is recruited to PIP2 at the plasma membrane. This causes a conformational change in STE5 to transition it to an open state such that FUS3 can bind to STE5. Upon binding to STE5, FUS3 then undergoes a conformational change to be properly presented to STE7 for phosphorylation.

In the simple model, STE5 binds to STE11, STE7, and FUS3, which are sequential kinases that transmit a signal from the upstream mating receptor STE2 to downstream transcription factors including STE12 (13) (Fig. 1, left). Thus, STE5 tethers components of a single pathway by simultaneously binding three kinases in a tiered cascade that is essential for a MAPK-like pathway (12) and thereby brings these kinases into proximity to enhance the efficiency of signal transduction (14, 15). STE5 scaffolding is now known to be more complicated (Fig. 1, right), in that STE5 facilitates this pathway in ways beyond just kinase binding (12, 15, 16). Recently, it was discovered that FUS3 exists in an inactive conformation that weakly associates with STE5 and that STE5 induces a change in FUS3 such that it is open for activation by STE7 (Fig. 1, right) (17, 18). Zalatan *et al*. (18) used XRC to demonstrate that STE5 regulates the yeast mating response by means of a two-stage conformational change, wherein STE5 is in an autoinhibited, inactive conformation that has a weak binding affinity for FUS3. This inactive state is relieved upon membrane binding of STE5 during the induced mating response, which enables STE5 to bind tightly to FUS3 and for STE5 to induce a conformational change in FUS3 that relieves its inactive conformation such that FUS3 can be phosphorylated by STE7 (18). This was a seminal discovery in this field because it demonstrated (1) that a scaffold could serve as a decision maker of a pathway, based on upstream signals, and (2) that a scaffold could actively induce a transition in a binding partner to impart fine tuning of pathway signaling.

### KSR: The RAS-RAF-MEK pathway

The mammalian RAS pathway is involved in a variety of cellular processes such as proliferation, apoptosis, migration, and differentiation (19). Activation of the RAS pathway leads to activation of a three-tiered kinase pathway consisting of RAF, MEK, and ERK. Much like STE5, kinase suppressor of RAS (KSR) proteins are human scaffolds that bind RAF (particularly B-RAF), MEK, and ERK and support this kinase signaling cascade that contributes to downstream cellular outcomes (20). Of note, KSR proteins are also essential for RAS-directed oncogenesis, consistent with their integral role in downstream RAF/MEK/ERK signaling (19, 21). KSRs appear to have evolved *via* convergent evolution with STE5 because KSR proteins and STE5 serve similar functions in their respective organisms but have no significant sequence similarity. While yeast have a single STE5-like protein, mammals have two KSR proteins (KSR1 and KSR2) that are mostly redundant in function and are structurally related to the RAF family of kinases (A-RAF, B-RAF, and c-RAF) (21, 22). Relevant to this review are the membrane targeting domain (CA3) and the C-terminal kinase/pseudokinase domain (CA5/KSR^KD^), due to the conformational control that these regions impart. While the kinase domain of RAF proteins is critical for downstream phosphorylation of MEK1, the activity of the pseudokinase domain of KSR continues to be a subject of debate, hence its classification as a pseudokinase.

**Figure 2:**
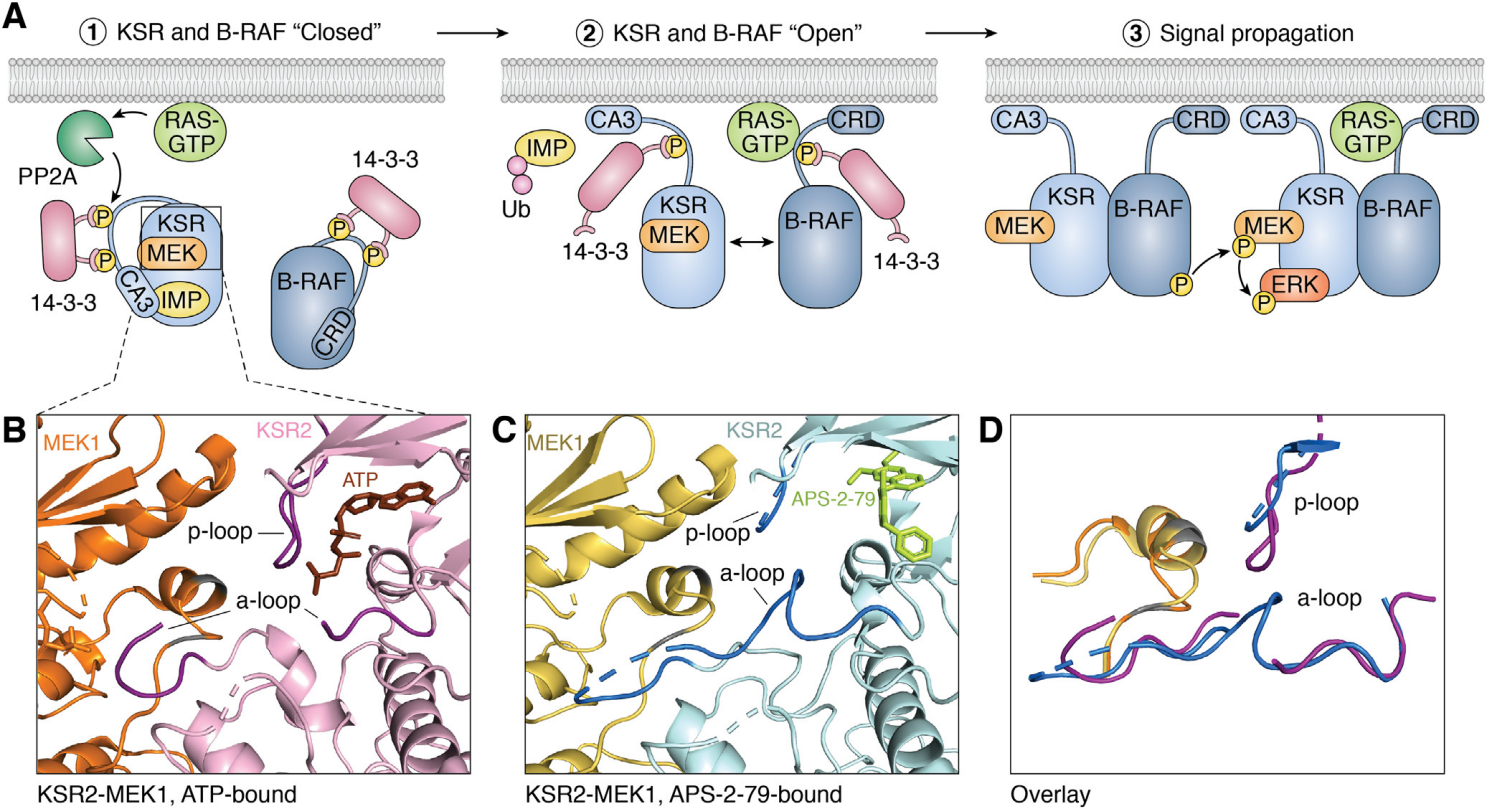
**Conformational change in KSR during RAS signaling.***A*, in the human MAPK pathway, the KSR–MEK complex exists in a closed conformation in the cytosol. Upon activation of the pathway, PP2A is recruited by RAS to dephosphorylate KSR in order to release its inhibition by 14-3-3 and IMP (1). The release of 14-3-3 and IMP causes a conformational change in KSR such that its CA3 domain can be tethered to the plasma membrane and KSR can dimerize with active B-RAF (2). Upon binding active B-RAF, KSR undergoes a second conformational change to present MEK for phosphorylation *in trans via* a separate KSR/B-RAF complex for signal propagation (3). *B*, view of the crystal structure of KSR2-MEK1 bound to ATP (*brown*) (PDB 2Y4I). Activation loop and p-loop (*dark purple*) are “closed” surrounding MEK1 218/222 (*gray*), reflecting the inactive RAF-inaccessible state of the complex. *C*, view of the crystal structure of KSR2-MEK1 bound to APS-2-79 (*light green*) (PDB 5KKR). Activation loop and p-loop (*dark blue*) are “closed” in a different state, reflecting another inactive, RAF-inaccessible conformation of the resting state. *D*, simplified superposition of the difference in the KSR active site bound to APS-2-79 (*dark blue*) and ATP (*dark purple*), centered around MEK 218/222 (*gray*), demonstrating the conformational flexibility of these segments in inactive complexes. Crystal structure images adapted from Dhawan *et al*. (29) and Brennan *et al*. (23). Images compiled in PyMOL 2.5. A-loop, activation loop; PDB, Protein Data Bank.

Due to the difficulty of crystallizing the full-length complex, no structure of an active RAF-KSR-MEK complex has been determined. However, several studies have used XRC to determine the structure of an inactive KSR–MEK complex. In one study, the crystal structure of KSR2^KD^ and MEK1 indicated that it exists in a closed conformation such that their catalytic sites are “face-to-face” and the primary activating phosphorylation sites on MEK1 (Ser218 and Ser222) are shielded from phosphorylation (23). Predictive modeling of the KSR2^KD^-MEK1 and the B-RAF crystal structures revealed that in order for the binding interface between KSR and B-RAF to align, a shift in an α-helix of KSR (termed αC) would be required. Functionally, this conformational change is accompanied by a shift in the activation loop of KSR, proximal to the ATP-binding pocket, which exposes Ser218 and Ser222 on MEK1 for phosphorylation by B-RAF *in trans* (Fig. 2*A*). However, lacking structural data of this active B-RAF-KSR-MEK complex, this shift of αC and the activation loop must still be validated. Thus, KSR enables the phosphorylation of MEK1 through a dimer-induced conformational change. Consistent with this model, several mutations in key B-RAF–binding sites in the pseudokinase domain of KSR (P662L, R684C, and R718H) have been predicted by XRC of the KSR^KD^ to eliminate key electrostatic interactions between KSR2 and B-RAF (23, 24). Other inactivating mutations in KSR, such as the I801L, G816D, R818Q, and R823H, are located within or near the activation loop of KSR and are therefore near to Ser218/Ser222 on MEK (24). These mutations in KSR likely impair the conformational change required to expose Ser218/Ser222 on MEK1 for phosphorylation by B-RAF.

Recent structural analysis by cryo-EM of B-RAF supports a second predicted conformational change in KSR, wherein the CA3 domain is exposed for membrane binding (25, 26). Based on what has been shown structurally for B-RAF (26) and biochemical analyses of KSR (27, 28), KSR likely undergoes a conformational change when dephosphorylated by protein phosphatase 2A at a critical 14-3-3 binding site, Ser392. This conformational change then exposes key residues in the KSR CA3 domain that promote its membrane association, which is required for RAS signaling.

The understanding of KSR2’s conformational changes required for B-RAF/RAS signaling suggested a method for locking KSR2 in an inactive conformation and thus blocking downstream RAS signaling. Indeed, Dhawan *et al*. (29) identified a small molecule (APS-2-79) that blocks RAS signaling by stabilizing the inactive form of KSR2. In that study, it was shown by XRC that APS-2-79 intercalates into the ATP-binding pocket of KSR2 forming tight interactions with the aromatic groups of Phe725, Tyr714, and Phe804. This fixes the closed “face-to-face” conformation of KSR2 such that Ser218 and Ser222 of MEK1 remain buried and unavailable for phosphorylation by B-RAF. They further showed that APS-2-79 blocks the interaction of KSR2 and B-RAF, presumably by preventing the conformational change in the αC helix *via* an “induced lock,” mimicking the inactive KSR conformation (Fig. 2, *B* and *C*). It is important to note, however, that the conformation of the activation loop of KSR is altered by APS-2-79, which further demonstrates the flexibility of this region (Fig. 2*D*). This finding, along with crystal structures of other inhibitors (30), supports the hypothesis that the activation loop in KSR is subject to regulation by conformational change. That is, APS-2-79 and other inhibitors appear to prevent KSR2 from adopting the open state and binding to B-RAF, thus providing a method to reduce the constitutive RAS activity found in RAS-driven cancers. Indeed, combined treatment of K-RAS mutant tumor cell lines with APS-2-79 and the MEK inhibitor trametinib decreases cell viability in a synergistic manner (29). Thus, APS-2-79 demonstrates the therapeutic value of mechanistic studies of conformational change in scaffold function.

### NEMO: The NF-κB pathway

NEMO (aka IKKγ) is a 419 amino acid scaffold in the transcription factor NF-κB pathway (31), which is involved in a variety of immune cell functions. NEMO is essential for canonical NF-κB signaling, as cells deficient in NEMO fail to activate the NF-κB pathway in response to multiple stimuli such as TNFα and IL-1β. Specifically, NEMO facilitates the ability of IKKβ to phosphorylate the NF-κB inhibitor IκB, which is necessary for the degradation of IκB to allow nuclear translocation and DNA binding by NF-κB. NEMO also provides an example of how conformational change in a scaffold can affect downstream signaling. Full or partial loss-of-function mutations in NEMO, which abrogate NF-κB signaling, have been found in a variety of human immunodeficiency diseases (31, 32).

**Figure 3:**
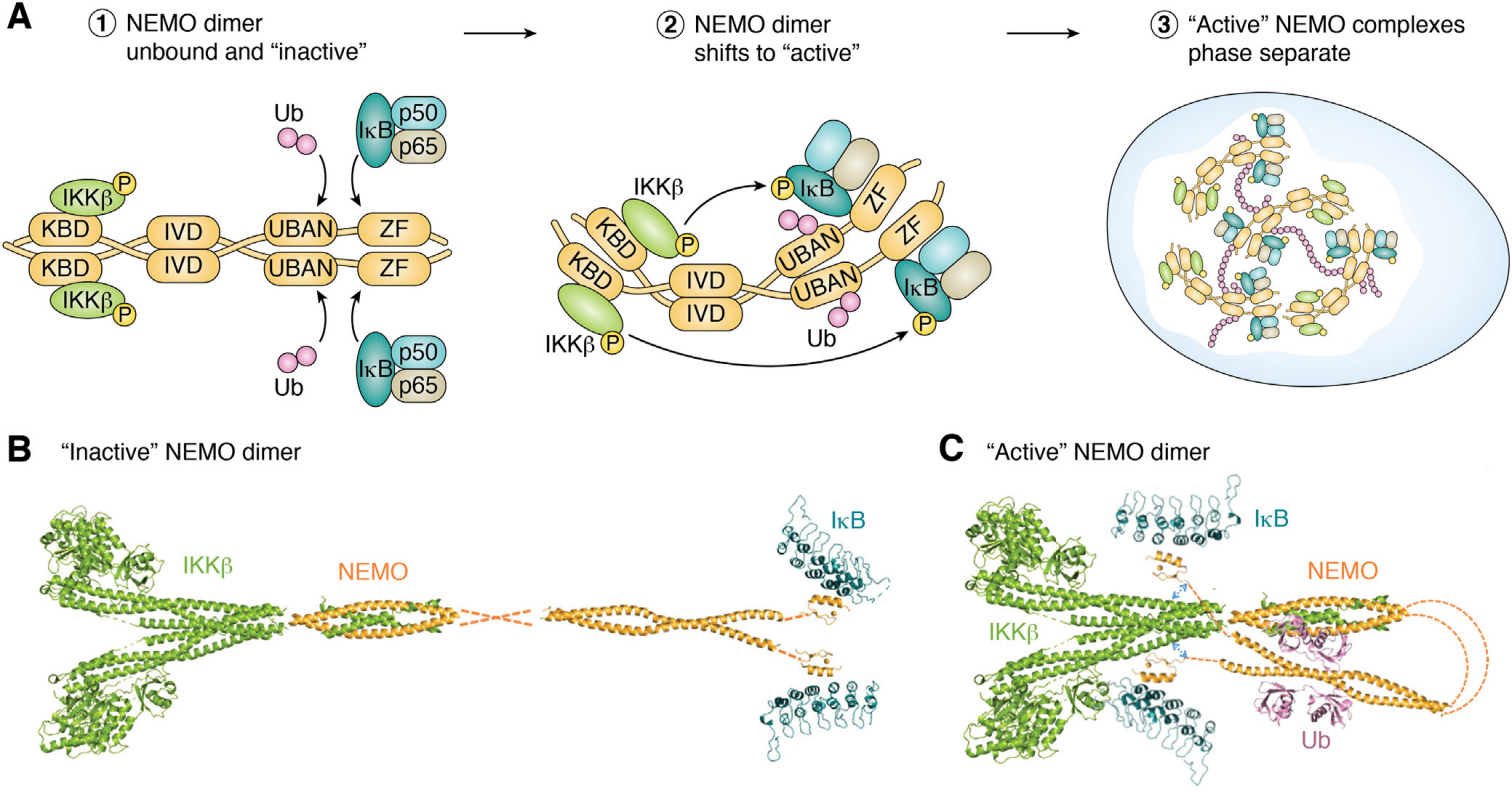
**Conformational changes in NEMO during NF-κB signaling.***A*, dimerized NEMO exists as an inactive extended coiled coil in the cytosol (1). Once it binds ubiquitin at its UBAN region and IκB at its zinc finger, NEMO undergoes a series of conformational changes to present a bound IκB to IKKβ for phosphorylation (2). This conformational shift in NEMO, induced by ubiquitination, promotes liquid–liquid phase separation of NEMO and its associated client proteins for efficient signaling (3). (p50 and p65 represent NF-κB, which is bound to IκB.) *B*, schematic representation of the inactive IKK complex, IKKβ (PBD 4KIK) is in *green*, NEMO KBD and IKKβ NEMO binding domain (PDB 3BRV); NEMO CoZi domain (PDB 2ZVO); NEMO ZF (2JVX) are in *orange*, and IκB (PDB 1IKN, p50/p65 omitted for simplicity) is in *dark cyan*. *Dotted orange lines* represent regions lacking structural data within NEMO. *C*, schematic representation of active IKK complex with conformational rearrangement centered around the central structurally uncharacterized region to bring IκB in proximity to IKKβ. *Blue dotted lines* with arrowheads represent the stabilization of the active conformation when NEMO residues 384 to 389 are bound to IKKβ, which is induced by the binding of linear ubiquitin (*pink*). Images compiled in PyMOL 2.5. PDB, Protein Data Bank; ZF, zinc finger.

As a noncatalytic scaffold of the IKK complex, multimeric forms of NEMO bind to a number of molecules including IKKβ, ubiquitin, LUBAC, and IκB in a high molecular weight complex of approximately 700 to 900 kDa (33, 34, 35, 36, 37). Several studies have demonstrated that IKKβ binds to the N terminus of NEMO at its kinase-binding domain (aa 44–110) (38), while the IKKβ substrate, IκB, binds to the C-terminal zinc finger (ZF; aa 389–410) domain of NEMO (36). Meanwhile, ubiquitin (primarily M1-linear ubiquitin) binds to the UBAN domain of NEMO (aa 289–320) (39). Due to its propensity to aggregate when expressed in bacteria, a crystal structure of full-length NEMO has yet to be solved; however, analytical ultracentrifugation of NEMO aa 1 to 355 (40) and SAXS of full-length NEMO in solution (41) have shown that unliganded NEMO is an extended coiled coil (Fig. 3, *A* and *B*), suggesting that NEMO must undergo structural rearrangement to bring N-terminally bound IKK and C-terminally bound IκB into proximity for the phosphorylation of IκB. However, there is no experimentally determined full-length molecular structure of NEMO, either alone or in complex with partner proteins, to confirm this hypothesis.

Nevertheless, structural studies of NEMO in solution have shown that it can undergo conformational changes upon ligand binding (Fig. 3*A*). For example, biophysical studies of full-length NEMO indicate that NEMO undergoes conformational changes following binding to IKKβ or linear ubiquitin (41, 42, 43). Specifically, it has been shown through 8-anilinonaphthalene-1-sulfonic acid/tryptophan fluorescence emission that incubation of NEMO with IκB and long ubiquitin chains induces a shift in emission of NEMO that is consistent with solvent exposure of hydrophobic residues (43). This, in turn, increases the affinity of NEMO for both the NEMO-binding domain of IKKβ and IκB, suggesting that NEMO undergoes structural rearrangement upon binding linear ubiquitin that increase its binding affinity for other substrates (43). This is corroborated by a recent study of NEMO using red edge excitation shift spectroscopy (44). By measuring the shift of the absorbance spectrum of NEMO bound to an IKKβ peptide, IκB, and different length ubiquitins, Catici *et. al*. (44) were able to study the conformational equilibrium of ligand-bound and ligand-unbound forms of NEMO. Their data demonstrated that NEMO’s conformational equilibrium changes upon ligand binding, especially when incubated with ubiquitin, demonstrating again that NEMO adopts discrete conformational states that enable it to interact with its primary binding partners IKKβ and IκB. Of note, some human disease mutations within the ubiquitin-binding domain of NEMO reduce its ability to activate IKK for downstream activation of NF-κB (33).

Ubiquitin-induced conformational change in NEMO has also been used to suggest that NEMO exists in an autoinhibited state that is relieved upon the binding of tetraubiquitin, due to the presence of a dynamic “hinge” region between the N-terminal IKKβ and C-terminal IκB binding domains of NEMO (42). For example, SAXS has indicated that NEMO adopts a more compact structure upon binding to IKKβ (41). Moreover, a synthetic mutation in a disordered central region of NEMO that eliminates the IKKβ-induced conformational change in NEMO also abolishes the ability of NEMO to support tumor necrosis factor–induced activation of IKKβ and downstream NF-κB in cells, demonstrating the necessity of conformational flexibility for the scaffolding function of NEMO (41). Finally, there is recent evidence that binding to linear ubiquitin induces the exposure of a second, weaker IKKβ-binding site in the NEMO ZF, which can stabilize the active NEMO signaling complex and bring IKKβ into proximity of IκB for phosphorylation (45). That is, a series of pull-down and mutation analyses indicated that a NEMO mutant lacking the canonical N-terminal IKK-binding domain was still able to interact with IKKβ following incubation with linear tetraubiquitin. Furthermore, aa 384 to 389 of human NEMO were identified as critical for this interaction with IKKβ, and this region of NEMO is immediately N-terminal to the proposed IκB binding site at the ZF, which would be exposed upon binding of linear tetraubiquitin (36, 45).

Taken together, the aforementioned data suggest that binding of IKKβ and ubiquitin elicits a series of conformational changes in NEMO that are required for NEMO to adopt the proper conformation to present IκB for phosphorylation by IKKβ (Fig. 2*B*). Not surprisingly, mutations in NEMO that disrupt IKKβ or ubiquitin binding disrupt its ability to function. Interestingly, several human disease mutations are in a central region of NEMO (termed the intervening domain [IVD]) that is not known to be required for the binding of protein substrates (31, 32). A synthetic mutation within the IVD has been demonstrated to affect the structural reorganization of NEMO that occurs in response to ligand binding (41). Thus, disease mutations in the IVD may impair NEMO by affecting its ability to undergo a conformational change necessary for IKKβ to phosphorylate IκB for downstream activation of NF-κB.

A recent study demonstrated how individual ligand-induced conformational changes in NEMO may have a larger scale effect on the NEMO signalosome (*i.e*., NEMO in a complex with ubiquitin, IKK, and IκBα). That is, Du *et al*. (46) showed that the binding of M1 and K63-linked ubiquitin to the NEMO UBAN and ZF regions elicits a LLPS of NEMO both *in vitro* and in cells. Ubiquitin-induced NEMO-containing LLPS droplets formed in response to stimulation with TNFα and IL1-β and were also enriched for phosphorylated IKKα/β and TAB, indicating that the droplets were required for NF-κB pathway activation. Thus, these phase transitions are likely linked to conformational changes in NEMO due to ubiquitin binding. Data from Catici *et. al.* (43) and Shaffer *et. al.* (41) support these findings, by their suggestions that structural rearrangement of the NEMO dimer exposes binding surfaces for protein–protein interactions, providing a modular multivalent platform in which LLPS occurs rapidly in a NEMO-nucleated signalosome (containing also IKK and IκBα) in response to NF-κB pathway activators.

### SHANK3: The postsynaptic density pathway

SHANK3 is a neuronal-specific scaffold found at the postsynaptic density (PSD) of excitatory synapses (47). SHANK3 appears to act as a master regulator of the PSD by organizing cytoskeletal elements to support synaptic signaling. SHANK3 has several protein-binding domains including, among others, an N-terminal SPN domain that binds RAP1 and actin during integrin signaling, an SH3 domain that binds to AMPA receptor (AMPAR) complexes, and an ANK domain that binds cytoskeletal elements such as α-fodrin (48, 49, 50, 51). Emerging evidence indicates that SHANK3 regulates the PSD, at least in part, through conformational changes that switch SHANK3 between active and inactive states based on its bound ligand (48, 52). Of note, loss-of-function mutations in SHANK3 are linked to neurodevelopmental disorders like autism spectrum disorder (49).

**Figure 4:**
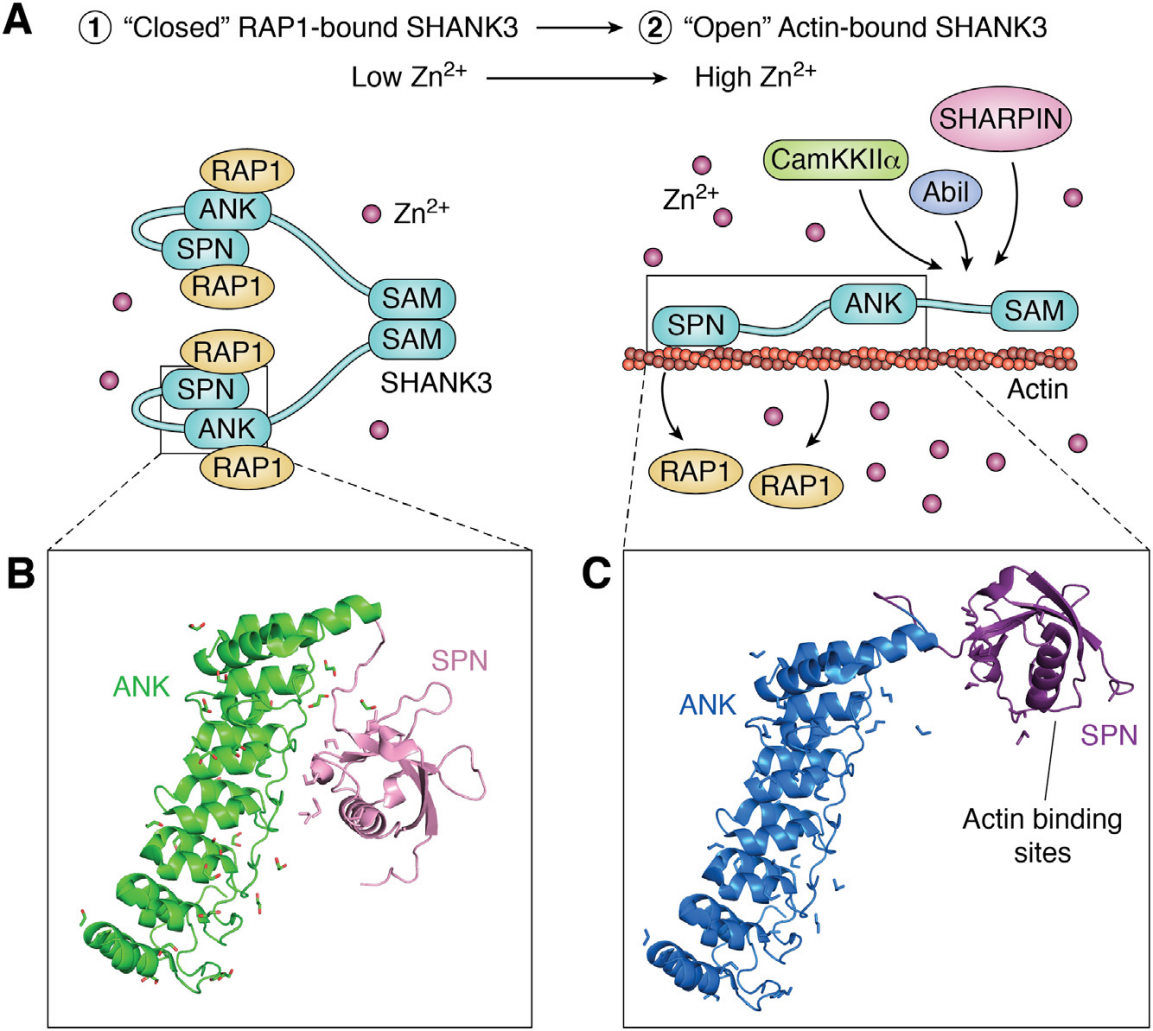
**Conformational change of SHANK3 during synaptic signaling.***A*, in the postsynaptic density (PSD), oligomerized SHANK3 is bound by RAP1 *via* its SPN and ANK domains, promoting its closed conformation. SHANK3 sequence C-terminal to the SAM domain omitted for simplicity (1). A signal, possibly an increase in zinc concentration at the PSD, causes a conformational change in SHANK3 to the open conformation. This causes RAP1 to be released from SHANK3, reduces the oligomeric state of SHANK3, promotes actin binding to SHANK3, and leads to the recruitment of actin regulators such as AbiI, CaMKKIIα, and SHARPIN to promote actin polymerization in the PSD (2). *B*, crystal structure of the SPN-ANK domain of SHANK3 (PDB 5G4X). The ANK domain is in *light green* and the SPN domain is in *pink*. Actin-binding sites are buried to prevent actin binding. *C*, schematic representation of the N52R mutant of SHANK3, which is constitutively in the open state, wherein RAP1 is unbound and actin-binding sites are exposed. The ANK domain is in *blue* and the SPN domain is in *purple*. Crystal structure images adapted from Salomaa *et al*. (48). Images compiled in PyMOL 2.5. PDB, Protein Data Bank.

Salomaa *et al*. (48) demonstrated a role for SHANK3 in the cytoskeletal organization of the PSD by modulation of its active (open) and inactive (closed) conformations (Fig. 4*A*). Based on the published crystal structure of the N-terminal SPN and ANK domains (53), they used molecular modeling to assess the effect of a point mutation (N52R) on the structure of the SPN-ANK domains. While the WT SPN and ANK domains are folded such that the SPN actin-binding residues are not exposed, the N52R mutant shows a clear shift in the linker region between the SPN and ANK domains. This shift then exposes the actin-binding sites of SHANK3 (Fig. 4, *B* and *C*). Salomaa *et al*. further showed that the functional consequence of the N52R mutation was to enable SHANK3 to bind more strongly to actin, suggesting that the N52R mutation mimics a biologically relevant conformational switch to an open form that regulates SHANK3 binding to actin at the PSD (48). In contrast, the closed conformation of SHANK3 is stabilized by the SPN domain binding to RAP1, leading to inhibition of integrin signaling (53). Release of this closed conformation of SHANK3 would therefore release RAP1 and stimulate integrin signaling. Interestingly, exposure of the ANK domain of SHANK3 leads to the recruitment and binding of a different cytoskeletal protein, α-fodrin (54).

Together, these data indicate that the conformational state of SHANK3 regulates an exchange between binding of its ligands in order to properly regulate cytoskeletal dynamics (48). Related to this model, two mutations in SHANK3 (R12C and L68P) linked to autism have been studied for their consequences on its scaffolding function (49). SAXS and thermal stability assays revealed that both autism mutations alter the overall structure, stability, and conformational flexibility of SHANK3. Furthermore, the R12C mutant does not persist in the synapse and the L68P mutant shows increased SHANK3 clustering, demonstrating two different modes of conformational instability for mutations within the SPN domain of SHANK3. Overall, studies of these mutants show that altering the conformational flexibility of SHANK3 results in a loss of its ability to regulate cytoskeletal clustering of PSD components, likely due to altered exposure of a protein-binding interface.

While the signal for the switch in SHANK3 binding to RAP1 *versus* actin is not yet known, a recent study (55) suggests that conformational control of SHANK3 is dependent, at least in part, on zinc levels within the PSD (Fig. 4*C*). That is, Arons *et al*. (55) described a conformational switch of SHANK3 that is dependent on zinc ion concentrations for the regulation of synaptic transmission at the PSD. Using FRAP, they showed that changes in zinc concentration in cells overexpressing SHANK3 affected the shuttling of SHANK3 within the PSD, thereby altering its oligomerization and the stabilization of the PSD signaling complex. They proposed that the zinc-dependent conformational switch from inactive to active SHANK3 allows for tighter binding to its PSD-associated partner HOMER. While the binding regions of HOMER and zinc are in the C-terminal proline-rich domain and SAM domain, respectively, it has been suggested that the SNP-ANK domain interacts with the C-terminal proline-rich domain, possibly linking these two conformational changes (56). Although there is no structural data to confirm this zinc-induced conformational change in SHANK3, these studies nonetheless demonstrate that a regulatory activity of SHANK3 is modulated by postsynaptic zinc concentration. Thus, this dynamic exchange of conformational states in SHANK3 is another example of active regulation of signaling by scaffold proteins.

## LLPS and scaffold assembly

While the information described above generally conforms to the traditional view of a scaffold, studies of LLPS have begun to use the term scaffold in a somewhat different manner. LLPS refers to the large-scale condensation of molecular components to catalyze a given biological process (57). The term “scaffold” in the field of LLPS is often used to designate a driver of LLPS, as connoting a self-associating biomolecule that interacts with various “clients,” that is, molecules that are recruited by interaction with their scaffold (58). The properties generally associated with LLPS drivers are multivalency, the presence of intrinsically disordered regions (IDRs), and an ability to assemble clients into membraneless compartments through a phase transition (59). Based on these properties, LLPS drivers are often involved in intracellular signaling but can also be involved in a variety of other processes that involve the assembly of protein complexes (60). Overall, the shared characteristics of LLPS drivers and signaling scaffolds suggest that their functions are, at least sometimes, one and the same. In both contexts, the proteins termed scaffolds bring their binding partners to the right place at the right time and in the correct biochemical context for optimal efficiency of a reaction.

As discussed above, dynamic conformational change, flexibility, and disorder are important for the activity of the signaling scaffolds STE5, KSR, NEMO, and SHANK3. Likewise, it is proposed that IDRs allow for conformational flexibility in LLPS-forming scaffolds that can be stabilized upon ligand binding. One example is the flexible protein HP1α (heterochromatin protein 1α), in which a conformational switch exposes hydrophobic residues to enhance protein–protein interactions that facilitate LLPS for heterochromatin formation and gene silencing (61). Thus, LLPS scaffold regulation is also dynamic by virtue of transient multivalent interactions that are modulated by conformational changes, similar to what we described previously for traditional signaling scaffolds.

Indeed, several signaling scaffolds have been demonstrated to drive LLPS through conformational plasticity, either directly or through their extensive disorder (Table 2). The presence of IDRs in LLPS scaffolds implies conformational plasticity in these molecules during droplet formation (62), and several scaffolds have been proposed to undergo conformational switching upon phase separation. Signaling scaffolds that have been shown to drive LLPS include the following: (1) PSD-95 acting in conjunction with SynGAP within the PSD (5, 63, 64); (2) SHANK3 and CTTNBP2 forming zinc-dependent condensates at the synapse (65); (3) 14-3-3 proteins in the MAPK pathway (66); (4) SQSTM1, which is involved in many cell signaling events, including cell growth, differentiation, and inflammation, forming LLPS droplets *via* its interaction with polyubiquitin (67, 68, 69); and (5) NEMO phase separating in the presence of M1-linked and K63-linked polyubiquitin (46). The latter two examples suggest that ubiquitin binding is a common mechanism of LLPS induction that is used by several scaffolds.

**Table 2:** Examples of scaffolds that undergo liquid–liquid phase separation in cellular signaling

Scaffold	Signaling pathway	Reference
NEMO	NF-κB	(46)
CIN85	BCR	(129)
SLP-65	BCR	(130)
LAT	TCR	(131, 132)
CTTNBP2	Synaptic pathway	(65)
PSD-95	Synaptic pathway	(64)
SHANK3	Synaptic pathway	(65)
APC	Wnt/β-catenin	(133, 134)
Axin	Wnt/β-catenin	(133, 135)
TAZ	Hippo	(136)
RIα	cAMP-PKA	(137, 138)
p62	Autophagy	(67)
NCK1	Actin polymerization	(139)

In any case, for many scaffolds there may be a blending of the terms dynamic scaffold and LLPS driver as more studies uncover mechanisms used by active signaling scaffolds. An exciting new tool to study conformational dynamics, recently developed by Harroun *et al*. (70), involves the attachment of fluorescent nanoantenna to a protein in a less functionally invasive way than traditional methods. This newly developed technique is similar to previously described methods that conjugate fluorescent dyes, such as Alexa488 and 594 to proteins, with the exception of utilizing a “nanoantenna” consisting of biotin, a linker region, and a fluorescent dye. In their study, Harroun *et al*. (70) used streptavidin (due to its extensive biotin-binding sites) to probe five distinct conformational states of biotinylated alkaline phosphatase, and they claim that the technique should be translatable to other biotinylated proteins. The advantage of this method over other conjugation techniques is the relatively low invasiveness of biotinylation on protein function and the ease of biotinylation with commercially available kits. Technical advances such as this may pave the way for the discovery of conformational changes in additional scaffolds, as well as a more precise understanding of how conformational change plays a role in scaffold function.

## Synthetic scaffolds as dynamic regulators

Several studies have used synthetic scaffolds made from the binding domains of natural scaffolds to further investigate signaling dynamics (71, 72). These synthetic scaffolds have provided a great deal of information for understanding signaling mechanisms by modifying chimeric signaling pathways, enhancing metabolic interactions, and understanding LLPS, specifically with regard to multimeric higher order complex formation. The synthetic optimization of natural scaffolds is analogous to what has been done in the field of “designer enzymes,” in which the main purpose is to optimize enzymatic reactions from natural enzymes (73). For designer enzymes, a natural enzyme is mutated such that it is more catalytically active and/or more stable than its natural counterpart (74). Similarly, the generation of designer scaffolds from natural ones to regulate or change signaling cascades could provide an enhanced understanding of how scaffolds have evolved and their roles in increasing the efficiency of signaling. Toward that end, designed variants of STE5 have demonstrated how engineered scaffolds can lead to a better understanding of the yeast mating pathway, including rewiring the mating response for unique outputs (1, 75). However, the challenge for designer scaffolds lies in modulating not only their interactions with binding partners but also how they alter the dynamics of signaling through conformational changes. Greater knowledge of the plasticity of scaffolds through tuning or optimization studies should allow for a deeper understanding of biological and biophysical processes.

## Conclusions and perspectives

Although scaffolds were once considered as stable platforms for reaction assembly, it is now clear that they play dynamic and complex roles in signaling by undergoing conformational changes (5, 76, 77). The dynamic properties of scaffolds can be required for regulation of the timing of signaling and to bring their molecular partners into the correct configurations and concentrations. The dynamics of the four scaffolds (STE5, KSR, NEMO, and SHANK3) reviewed herein highlight the importance of structural rearrangements within a scaffold for proper function. Not surprisingly, ablation of a scaffold’s essential conformational change can be detrimental to a given signaling pathway, as mutations in scaffolds that prevent such changes can prevent the proper assemblage of binding partners on the scaffold. An understanding of these types of rearrangements is crucial for developing a full picture of scaffold activity and to capitalize on the potential to develop synthetic and therapeutic molecules to modulate scaffold activity. Indeed, four studies have shown that small molecules that trigger conformational changes in scaffolds or lock them in improper conformations may be a promising approach for treating certain cancers, inflammatory diseases, and Alzheimer’s disease (21, 78, 79, 80).

Overall, the designation of scaffolds as inert players in signaling, and other subcellular processes, is no longer valid. Based on emerging information, it is likely that all scaffolds undergo some form of conformational change that brings their molecular partners into proper configurations for biological outputs. In addition to the proteins discussed previously, many scaffolds listed in Table 1 undergo some form of an “on-off switch” or are otherwise regulated through general plasticity and flexibility, either in the framework of traditional signaling or *via* LLPS. This on-off switch paradigm or even more complex sets of conformational states stabilized by ligand binding appears to be a common mechanism among scaffolds and is reflective of the rapid regulatory capacity that scaffolds impart for cellular processes. Going forward, scaffolds should be viewed as dynamic and malleable integrators of multiprotein molecular processes.

## Conflict of interest

The authors declare that they have no conflicts of interest with the contents of this article.
